# Perspectives in the Scientific Literature on the Barriers and Benefits of the Transition to a Plant-Based Diet: A Bibliometric Analysis

**DOI:** 10.3390/foods14172942

**Published:** 2025-08-23

**Authors:** Lelia Voinea, Ana-Maria Badea, Răzvan Dina, Dorin Vicențiu Popescu, Mihaela Bucur, Teodor Mihai Negrea

**Affiliations:** The Faculty of Business and Tourism, The Bucharest University of Economic Studies, 41 Dacia Blvd., Sector 1, 010404 Bucharest, Romania; lelia.voinea@com.ase.ro (L.V.); dorin.popescu@com.ase.ro (D.V.P.); mihaela.bucur@com.ase.ro (M.B.); teodor.negrea@com.ase.ro (T.M.N.)

**Keywords:** plant-based diet, positive outcomes, obstacles, environmental sustainability, dietary transition

## Abstract

Plant-based diets are increasingly attracting attention as they play a significant role in human health and environmental sustainability and are believed to be key components of sustainable food systems. In the present study, both pros and cons of the adoption of plant-based diets are analyzed using a bibliometric method integrated with a qualitative examination of the scientific literature. For the bibliometric study, Bibliometrix software was utilized, examining 3245 scientific articles, downloaded from the Scopus database, and printed between the years 1957 and 2025. The analyses were conducted using R software, version 4.4.1, with access to the Bibliometrix package, version 4.1. The results indicate a remarkable rise, in the last two decades, in the scholarly focus on the influence of plant-based diets on the individual’s health condition as well as the environment. Keyword co-occurrence studies and international collaborations demonstrate a dominance of research focus in both the United States and Europe, with significant contributions from the Asia–Pacific region. Furthermore, the current work offers qualitative identification of the benefits of plant diets from various perspectives like nutritional, economic, ecological, and cultural. It also explores the main dissuaders from adhering to these diets, including perceived nutritional hazards, cost perceptions, low availability, and social prohibitions. Findings emphasize that, in spite of all the barriers, plant food-based diets have a wide-ranging ability to provide tangible benefits at both the individual and population levels, and documented in the scientific literature are recommendations of expert-led education programs, economic incentives, and judiciously framed public policies to overcome these barriers and to make this transition possible towards sustainable food choices. Findings provide a comprehensive understanding of the current lines of inquiry and stage the subsequent work on how to motivate sustainability among the general population.

## 1. Introduction

In recent years, plant-based diets have gained increasing popularity as viable approaches to improving human health, promoting animal welfare, and mitigating environmental impacts. Although vegetarian and vegan communities remain relatively small, they have experienced notable growth, particularly in Western societies [1]. While the terms “vegetarian” and “plant-based” are often confused, the latter encompasses a broader dietary spectrum that may include limited consumption of animal products such as milk, eggs, fish, and meat [2].

The transition to a predominantly plant-based diet presents significant challenges related to nutrition, sensory attributes, and food technology. Producers of plant-based alternatives face obstacles in mimicking the nutritional value and sensory experience of animal-based foods, making appropriate ingredient selection and food formulation essential [1]. For example, meat proteins are being replaced with protein isolates from various legume species [3,4,5]. Plant protein microgels are further used to improve the taste, aspect, and texture of these products, thereby increasing their sensory appeal [6]. Regarding plant-based milk alternatives, they are produced from various cereals, legumes, and nuts that are processed to mimic the appearance and texture of cow’s milk [7].

However, consumer acceptance remains a significant barrier, largely due to food neophobia and skepticism towards unfamiliar or highly processed plant-based alternatives, but also due to the sensory and cultural attachment to meat of a significant portion of the global population, especially in Western countries. Overcoming these perceptual barriers requires early exposure to diverse foods and sustained educational efforts [8,9,10].

The environmental benefits of plant-based diets are highly debated in the scientific literature, particularly in terms of their potential to reduce greenhouse gas emissions, water use, and land degradation, which represent key issues in global sustainability [6,11,12,13,14,15]. In terms of health benefits, the most frequently reported include reduced risks of cardiovascular disease and certain cancers due to reduced intake of saturated fat and cholesterol [5,16,17,18,19].

As consumers shift towards sustainable and health-oriented diets increases, the plant-based food sector is expected to expand [20]. To ensure widespread adoption, coordinated efforts in product innovation, marketing, and public education are essential to reshape preferences and overcome resistance [21,22]. Despite the growing academic interest in the impact of plant-based diets on health and the environment, we believe that investigating the benefits and barriers to transitioning to a plant-based diet from a complex perspective, integrating nutritional, economic, environmental, and cultural aspects, requires increased attention in the future. To fill this gap, a bibliometric analysis and a qualitative assessment of the scientific literature were carried out, with the main aim to trace the development of scientific research on plant-based foods and highlight the perceived impact of plant-based diets, in terms of nutritional adequacy, environmental sustainability, economic conditions or cultural values.

Thus, the main objectives of our study are as follows: to perform a comprehensive analysis of the evolution of scientific interest on plant-based diets to uncover thematic patterns, international research collaboration and publication dynamics from 1957 to 2025 and to identify the nutritional, economic, ecological and cultural benefits of plant-based diets, as well as the main perceived barriers to their adoption.

The results obtained will allow the development of evidence-based recommendations for educational initiatives and public policy interventions that support the transition to sustainable food systems by increasing the acceptability and adoption of plant-based diets.

The study begins with an introductory section providing some background elements and presenting the general framework of plant-based diet. Items of previous research in the area are also discussed in this section. The next section is dedicated to the presentation of the methodological approach, emphasizing the steps followed in conducting the study. While the third section includes the presentation of the results, the last one includes the conclusions of the study.

## 2. Literature Review

### 2.1. Historical Context of Plant-Based Diets in Research

The scientific discourse surrounding plant-based diets has evolved considerably since the mid-20th century. While initial studies mainly framed such dietary patterns in terms of religious, ethical, or philosophical beliefs [23,24], later research has moved toward exploring the broader implications of plant-based diets for public health, environmental sustainability, and food system resilience [25,26]. The steady increase in the number of publications over the past few decades reflects a shift in how plant-based diets are understood: not just as individual choices for a healthy lifestyle, but as essential components of sustainable food system development strategies [27,28].

Plant-based diets involve a nutrient-rich diet with a low intake of animal-based foods. Therefore, finding substitutes for animal foods is a difficult task for producers. Through innovative technology, meat proteins are replaced with those derived from legumes (such as soy, peas, chickpeas, lentils), lupins, wheat gluten, and mushrooms [3,4]. Plant protein micro-gels are further used to improve the taste, aspect, and texture of these products, thereby increasing their sensory appeal [6]. But not only can meat be replaced with plant-based substitutes, even milk can achieve plant-based status, often using water-soluble extracts from cereals (oats or rice), legumes (soybeans), and nuts (almonds and coconuts). The liquids are thus obtained by breaking down the extracted plant material in water and homogenizing it, so that, in the end, they mimic milk in both appearance and consistency [7].

### 2.2. Nutritional and Health Issues of Plant-Based Diets

Health outcome studies on plant-based diets highlights significant health benefits, particularly in improving lipid profiles and cardiovascular health and potentially reduce the risk of certain cancers [5,10,17,18,19,29,30,31]. For example, Craig and Mangels [29] provided extensive epidemiological and clinical data through their study showing that well-planned vegetarian diets are associated with lower LDL cholesterol, lower blood pressure, and reduced incidence of ischemic heart disease. These benefits are attributed to higher intakes of fiber, unsaturated fat, and antioxidants, as well as lower saturated fat and heme iron. These findings are confirmed by those of Richter et al. [17], who showed that plant proteins, especially when consumed with fiber-rich foods, reduce total and LDL cholesterol, highlighting the importance of the protein source in reducing cardiovascular risk. In the Romanian context, Petrescu-Mag et al. [5], using a causal network model, showed that meat substitutes can contribute to improved health, identifying cardiovascular disease risk reduction as a key motivation for consumers, integrated into broader systemic and policy influences. However, a nuance was introduced by the study by Tso and Forde [30], who cautioned that although switching to plant-based diets generally reduces saturated fat and cholesterol intake, nutritional outcomes vary depending on whether the diet is based on whole foods or ultra-processed alternatives.

These products, typically rich in fiber, phytochemicals and antioxidants, are also linked by epidemiological and metabolic studies to lower body mass index (BMI) and improved metabolic markers, as insulin sensitivity and blood pressure [32,33,34]. In a review of clinical and observational studies, Craig [32] found a consistent trend for lower mean body mass index (BMI) among vegetarians compared to omnivores. This effect is attributed to the higher consumption of high-fiber, low-energy-density foods, such as fruits, vegetables, and whole grains, which increase satiety and reduce overall caloric intake. Craig [32] also found improved glycemic control and insulin sensitivity in vegetarian populations. Farmer et al. [33] reinforces these findings through a cross-sectional analysis of National Health and Nutrition Examination Survey (NHANES) data from 1999 to 2004 and shows that vegetarian dietary patterns are associated with increased nutrient density and significantly lower BMI and waist circumference, thus positioning plant-based diets as a viable approach for long-term weight management. Building on this, Bowman [28], using NHANES data from 2013 to 2016, shows that vegetarian diets are correlated with reduced intake of calories, saturated fat, and sodium, along with higher consumption of whole plant foods, confirming the link between plant-based diets and improved metabolic markers.

Although numerous studies acknowledges that plant-based diets have higher intakes of dietary fiber, magnesium, folate, vitamins C and E [35,36,37,38], some studies have raised concerns about potential nutritional deficiencies, particularly of vitamin B12, vitamin D, iron, zinc, iodine, calcium, and omega-3 fatty acids, which require additional supplementation [39,40,41]. For example, Melina et al. [39] caution that such diets must be carefully structured to address specific micronutrient vulnerabilities. Nutrients commonly identified as potentially inadequate include vitamin B12, vitamin D, long-chain omega-3 fatty acids (EPA and DHA), iron, zinc, iodine, and calcium. Complementing this perspective, Neufingerl and Eilander [40] present a systematic review comparing nutrient intakes and biomarkers in individuals following plant-based versus omnivorous diets, which reveals consistent deficiencies in micronutrients such as vitamin B12, vitamin D, calcium, iodine, and selenium in plant-based populations. The study of White [41], focusing only on vitamin B12 intake, shows that its deficiency in people with a predominantly plant-based diet is high, even among vegetarians who consume dairy, eggs, or both. The authors mentioned above suggest that education on fortification, supplementation, and dietary diversification is essential, especially for vulnerable groups such as children, pregnant women, and the elderly. Concerns have also been expressed about the allergenic potential of plant-based products, especially those formulated with ingredients such as legumes (especially soy) or nuts, which are known to be common allergens among sensitive populations [42].

### 2.3. Environmental and Sustainability Aspects

The ratio of plant to animal protein in the diet is widely recognized as a key factor in ensuring the sustainability of food systems, with numerous studies indicating that a shift toward predominantly plant-based diets can offer significantly greater environmental and animal welfare benefits compared to diets high in animal protein consumption [6,11,12,13,14,15,43,44,45,46]. The common findings of these studies have shown that the production of plant proteins requires much fewer natural resources (water, agricultural land) while generating much lower greenhouse gas emissions compared to animal products, and also that these products offer an ethical alternative for consumers concerned about animal welfare.

However, some Life Cycle Assessment (LCA) studies demonstrates that the industrial-scale extraction, concentration, and purification of plant proteins require significant inputs of natural resources, energy, and chemical reagents, representing environmental costs that are frequently underestimated in conventional environmental impact assessments [47,48].

As highlighted by Yu et al. [49], the process of isolating proteins from plant matrices involves multiple operations, including mechanical grinding, extraction and precipitation, each of which contributes to the generation of a waste stream. Specifically, the use of organic solvents and chemical processing aids during the extraction process generates hazardous waste, presenting significant challenges in terms of environmental management. In addition, the findings of Peydayesh et al. [50] and Sproul et al. [51] indicate that the processing phase of plant protein production contributes significantly to greenhouse gas emissions, with each kilogram of protein waste being correlated with substantial releases of carbon dioxide into the atmosphere, thus exacerbating the impact of climate change. Mitigating these environmental burdens requires the development of more resource-efficient extraction methodologies and the implementation of circular economy principles by recovering waste and integrating it into alternative value chains [48,52].

### 2.4. Economic Dimension

From an economic standpoint, the transition toward predominantly plant-based diets holds the potential to support economic development by stimulating investment in innovative sectors such as alternative protein production and sustainable food technologies. Nevertheless, the economic affordability and accessibility of plant-based diets remain a subject of ongoing debate in the literature. For instance, the study of Masset et al. [53], Grabs [54], Pais et al. [55] and Viroli et al. [56] suggest that plant-based diets, particularly those based on minimally processed whole foods such as legumes, grains, fruits, and vegetables, can be cost-effective compared with typical omnivorous Western diets. In contrast, other studies, such as Fehér et al. [2], Lusk and Norwood [57], Bunge et al. [58], Rickerby and Green [59] highlight that the cost of plant-based diet may be elevated, particularly when it involves highly processed or specialty vegan products.

The emerging technologies such as cell culture, 3D printing, thermoplastic extrusion and enzymatic processing appear to be sustainable alternatives to minimize the production cost of plant-based products, increase scalability, and improve product quality. While cultured meat, produced through cell culture techniques, offers a resource-efficient and environmentally friendly alternative to animal farming [9,60,61], 3D printing enables precise patterning of plant-based products, minimizing material waste and energy consumption [62]. Enzymatic processing improves texture and flavor, aligning plant analogs more closely with traditional meat, while improving nutritional quality by reducing antinutrients [63,64]. Finally, thermoplastic extrusion enables the large-scale production of textured plant proteins, simulating meat-like structure in a cost-effective manner [65]. Together, these technological innovations can contribute to more sustainable food systems, supporting the economic viability and consumer appeal of plant-based foods, especially those in the category of meat substitute.

### 2.5. Barriers to Adoption of Plant-Based Diets

Despite widespread recognition of their benefits, however, previous research shows that the plant-based diets face several adoption barriers.

Firstly, food neophobia, defined as the unwillingness to taste new foods and the avoidance of unfamiliar foods [66,67], might represent a significant barrier to the adoption of plant-based foods. This reluctance towards new or unusual foods can reduce consumers’ openness to plant-based alternatives, even in the context of increased awareness of their health and environmental benefits [68,69].

Secondly, as demonstrated in prior research, perceived “naturalness” constitutes a critical determinant of consumer acceptance of novel foods [70,71]. This perception is shaped by several factors, including the origin of the product, the technological processes involved, the nature of the ingredients used, and the characteristics of the final product. Moreover, the studies of Butu et al. [72] and Bearth and Siegrist [73] underscore that consumers often exhibit strong negative emotional responses, such as fear or aversion, towards unfamiliar food technologies and products, which are frequently associated with the idea of “artificial” and “unnatural”. Although plant-based diets may be nutrient dense, consistent with dietary guidelines [33], from the perspective of the NOVA classification (NOVA comes from the Portuguese expression “nova classificação”, which translates as “new classification”) [74,75], most plant-based products, especially meat analogs, are classified as ultra-processed foods (UPF) because they often undergo extensive formulation and processing to improve the sensory and nutritional profiles. Thus, in the previous research [1,6,76,77,78] are highlighted a series of common characteristics of plant-based products, which show that the “plant-based” does not always designate a healthy product. For example, synthetic food additives are commonly incorporated, including preservatives aimed at inhibiting the growth of pathogenic microorganisms or flavor enhancers and bitter taste inhibitors aimed at improving unwanted flavors (often described as “astringent”, “grassy” or “sulfur”) from the specific plant source. In addition, when it is desired to obtain products that mimic the sensory attributes of conventional meat, the plant-derived ingredients are frequently subjected to industrial processing conditions that generate lipid oxidation to impart a flavor similar to natural meat and a higher protein oxidation than that which occurs in animal-derived meat. Furthermore, plant-based products are often fortified with synthetic micronutrients, most notably vitamin B12, iron, and zinc, to compensate for potential deficiencies in plant-based diets [79]. A notable concern is also the typically high salt of sodium content of these products [80].

Thirdly, the sensory as well as cultural attachment to meat, deeply rooted in many societies, especially Western ones, where meat is a mainstay of traditional diets, represents another major impediment to the adoption of plant-based meat alternatives [22,81,82]. In addition, as the study of Abbaspour et al. [21] and Liu et al. [83] showed, consumers often anticipate a reduced sensory value from plant-based alternatives, which hinders widespread acceptance and adoption.

Hence, in order for the transition to a largely plant-based food system to fulfill its potential for transformation, it is crucial that all obstacles to its path are met and addressed [16,22]. This dietary change holds tremendous potential not only in terms of making the environmental sustainability better, but also in improving the overall quality of life. Aside from supporting technological innovation in developing food substitutes, this transformation is also essentially involved in educating consumers, helping them to remodel their attitude and preferences.

### 2.6. Policy and Educational Interventions

Breaking through these obstacles effectively requires an all-embracing and multi-faceted approach, including educational interventions and public health strategies to facilitate the transition towards preponderantly plant-based diets. Given that previous efforts to promote sustainable food consumption, such as consumer education, informative product packaging and intervention campaigns towards restricting some diet-related behaviors, have been inadequately successful, a more multidisciplinary and extensive approach should be used in synchronization with the actual mechanisms that guide consumer food purchasing. As consumers at large would normally favor cost, taste and quality to the question of sustainability, effective interventions would have to aim at these key determinants and involve the principal stakeholders along the food chain of players, e.g., producers, food industry companies and retail chains [84].

One particularly impactful strategy involves revising national dietary guidelines to explicitly incorporate sustainability criteria, as such updates can contribute to improving public health outcomes and reducing environmental impacts, as outlined by Jacobs et al. [25] and Garnett et al. [85]. National dietary guidelines function as powerful policy tools whose evidence-based nutritional recommendations are aligned with public health and environmental sustainability goals, thereby encouraging increased consumption of plant-based foods. Also, academic programs and mass media campaigns have shown potential in changing consumer perceptions and attitudes and drive greater acceptance of plant-based alternatives [86,87,88].

### 2.7. Methodological Justification and Study Contribution

Bibliometric approaches have emerged as valuable tools for mapping the evolution of research in the field of sustainable diets. While some studies have used bibliometric analysis to explore broader themes in nutrition and sustainability [89,90,91,92], relatively few have focused specifically on plant-based diets [93,94]. This gap justifies the present study’s approach, which combines bibliometric data with qualitative review to provide a more holistic understanding of thematic development across time and regions.

## 3. Research Methodology

This research sought to highlight the main concerns of research when analyzing the literature on plant-based food consumption through a quantitative bibliometric analysis. The main advantages and barriers of consuming plant-based alternatives were additionally accentuated via a qualitative examination of the literature.

Bibliometric analysis of scientific articles in economics is a quantitative method used to assess and map the scientific literature in this field. Within this methodology, bibliographic data pertaining to publications, citations, authors, and institutions are carefully collected and analyzed so as to discern patterns, developments, and influence exerted by research throughout a designated domain. It aids comprehension of how economic inquiry has developed and impacted our world across time while pinpointing contributors and nascent avenues of study [95,96]. This evaluation of scientific production lends support to research orientations via deliberate planning, whilst pinpointing deficiencies within the literature [97].

Bibliometrix software allows the conversion of textual data into numerical data, which facilitates bibliometric analysis in this study. The study utilized the Scopus database for the compilation of bibliographic data. The selection of Scopus was motivated by criteria such as its extensive interdisciplinary coverage, rigorous indexing standards, and advanced bibliometric functionalities, which render it particularly suitable for conducting bibliometric analyses [98]. Moreover, Scopus provides access to a wide range of journals in the social sciences and other interdisciplinary domains that are highly relevant to the topic of plant-based diets, which lie at the intersection of health, environmental sustainability, nutrition, and policy.

The database accessible through the Scopus platform was explored in April 2025 and identified 3245 scientific articles on the subject of plant-based diets for consumers. Initially, the Scopus database identified 223,171 articles. Subsequently, filters selected only scientific articles published in journals. Fields relevant to our research were retained, and fields such as psychology, energy, engineering, art, etc., were eliminated.

In order to pinpoint the most applicable works, investigators employed a compilation of search terms: (‘plant-based diet’ OR ‘vegetarian diet’ OR ‘vegan diet’ OR ‘plant-forward diet’ OR ‘flexitarian diet’) AND (‘food consumption’ OR ‘dietary patterns’ OR ‘eating habits’ OR ‘nutrition’ OR ‘nutritional quality’ OR ‘sensory quality’ OR ‘disease prevention’ OR ‘environmental impact’ OR sustainability OR barriers OR benefits). The span that was analyzed occurred between 1957 and 2025, to highlight the evolution of citizen interest over decades.

Following the bibliometric analysis, a qualitative assessment of the scientific literature was carried out, which allowed a detailed analysis of the benefits and barriers associated with the consumption of plant-based foods as reflected in the literature. This second stage included an extensive sample of articles, selected not only from Scopus but also from other databases (Google Scholar, PubMed) in order to enrich the perspective on the topic by integrating recent, relevant and multidisciplinary contributions that were not fully captured in the bibliometric analysis. The qualitative analysis aimed to extract and synthesize key aspects on the nutritional, economic, environmental and cultural benefits of plant-based diets, as well as to identify the main perceived barriers to their adoption. The combination of the two approaches, quantitative and qualitative, provided a coherent and complex overview of the evolution of research in the field, capturing both the quantitative trends in scientific output and the interpretative depth of the content analyzed.

## 4. Results and Discussion

The analysis led to the identification of 3245 scientific articles relevant to the consumption of plant-based foods. The considerable volume of papers reflects the extent and growing interest of researchers in this topic, particularly in the last two decades, as shown in Table 1.

Between 1957 and 1990, research was few, merely 122 articles, representing 3.75% of total publications. This value suggests a certain nascent or rather restricted interest regarding the study of consumer behavior with respect to plant-based products. From 2006 to 2015, interest escalation pertaining to this topic becomes visibly improved, accounting for 14.20% of publications. Possessing approximately 20.24% of the published articles, the discipline shows a large augmentation spanning 2016 to 2020. This phenomenon is further heightened throughout 2021–2023 period, achieving the publications’ apex at 32.01%, mirroring continuous and common interest originating from the scientific community. A noticeable quantity of articles is indeed contained within the database, although the data pertaining to the period of 2024 until 2025 are partial, seeing as it was created during April 2025. A growth within the quantity of articles is anticipated by 2025’s conclusion.

**Figure 1:**
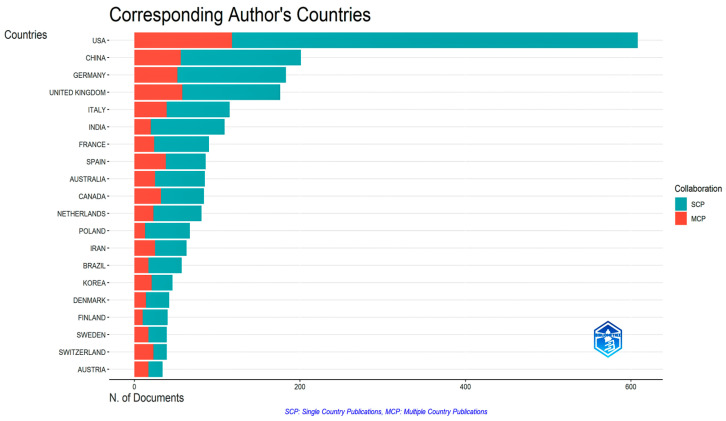
Country distribution of corresponding authors and typology of scientific collaborations (SCP vs. MCP). Source: Bibliometrix author data processing.

In terms of the most widely disseminated data (Figure 1), the United States (US) is in the lead, with over 600 articles published, demonstrating persistent research in the field of plant-based products. This focus is mainly corroborated by national publications (SCP) indicating substantial research capacity at the national level. China is followed by Germany and the United Kingdom, with a relative balance between domestic and international collaborations, along with a significant number of different publications each. Nordic countries such as Denmark, Finland, Sweden, and Switzerland have a high proportion of international collaborations (MCP). This implies that they gravitate tactically towards cross-border scientific partnerships, despite the low number of publications.

Furthermore, based on data processed by the authors using Bibliometrix and Web of Science, an analysis was carried out to identify the leading authors in this field, based on indicators such as the H-index, total number of citations, number of publications, and institutional affiliation. This analysis, which resulted in a ranking of the top 10 authors worldwide, once again highlights the dominant role of the United States, with six of the top ten authors, with Hu F. (H-index 254 and 245,446 citations) in first place. China, represented by authors such as Li Y. (H-index 78 and 25,631 citations), is growing in terms of individual scientific influence, although it still lags behind the American leaders. The United Kingdom and Germany are also represented by established researchers, such as Fraser G.E. (H-index 72 and 23,764 citations) and Leitzmann C. (H-index 26 and 2325 citations), respectively. Analysis of the most widely disseminated data, corroborated that of the most prolific authors, highlighting not only the volume but also the quality of research results, shows that the US maintains a clear position as world leader, followed by growing contributions from China, the United Kingdom, and Germany.

The United States, as a world leader in plant-based diet research, stands out with a substantial number of longitudinal studies, randomized controlled trials, and systematic reviews, along with influential position statements and meta-analyses from various academic and medical institutions supporting plant-based nutrition for disease prevention and treatment [18,99,100,101,102]. This great body of work has informed the inclusion of vegetarian dietary patterns in the 2020–2025 edition of the Dietary Guidelines for Americans [103]. Despite shaping international dietary discourse, however, research output does not align with adoption: US adoption of plant-based diets (25.8% in 2022) trails behind some European regions (up to 38%), suggesting that research leadership does not automatically translate into societal implementation [104]. Economic barriers, exacerbated by agricultural subsidies favoring animal products and cultural resistance are reported as key obstacles to adopting plant-based diets in the US [105,106].

However, emerging scholarly engagement from the Asia-Pacific region indicates a progressive internationalization of the discourse on food sustainability, reflecting a growing global awareness of the imperatives of the food transition.

**Figure 2:**
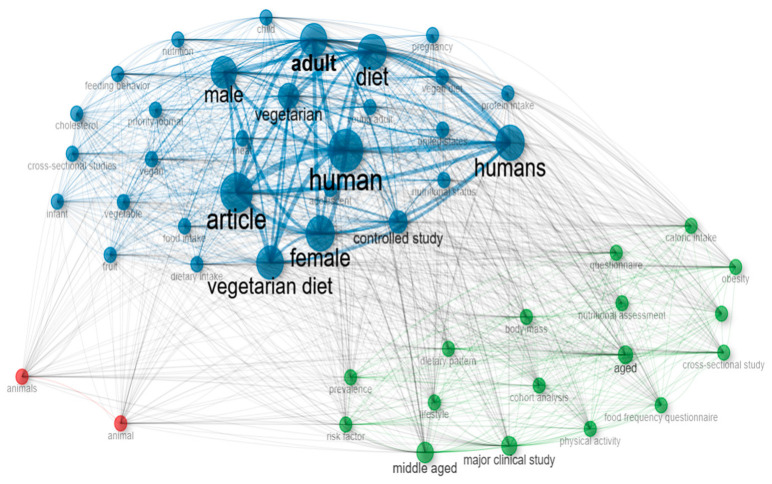
Keyword co-occurrence network in scientific literature on plant-based diets. Source: Bibliometrix author data processing. Colors indicate thematic clusters (blue, green, red), node size reflects keyword frequency, and lines represent co-occurrence links.

In terms of the meaningful vocabulary employed amid studies, three prominent groupings existed pertaining to plant-based diets (Figure 2). Blue nodes denote the most prominent and connected cluster, mirroring the major research concern regarding the adult human population, incorporating central terms such as “human”, “adult”, “male”, “female”, “vegetarian diet” and “diet”. The profusion of these designations implies that investigations stress evaluating vegetarian meal plan consequences upon grown people amid communal wellness. Dietary subtypes alongside dietary behaviors are denoted via interconnection alongside terms such as “vegan”, “vegetable”, “fruit”, and even “food intake”. “Dietary pattern”, “nutritional assessment”, “body mass”, “obesity”, “caloric intake”, and “cohort analysis” are designations that the green cluster collectively amalgamates. This group advocates assessing the way plant-based diets impact overall health, most notably with respect to body weight, obesity prevalence as well as caloric intake levels. The phrases “cross-sectional study”, “questionnaire” and “major clinical study” denote methodological precision. Observational studies as well as standardized data collection instruments are frequently employed, thereby reflecting this rigor. The red cluster, which includes the terms “animal” and “animals”, represents a thematically distinct group of studies focused on animal-related topics, particularly the consumption of animal-based products or the use of animal models. This cluster appears thematically and structurally isolated, with weak connections to the rest of the network. Its low frequency suggests that such topics are marginal within the analyzed literature, which predominantly centers on plant-based diets, human nutrition, and vegetarian dietary patterns. The focus of the scientific discourse is thus clearly shifted toward plant-based consumption, while animal product consumption is less commonly addressed.

**Figure 3:**
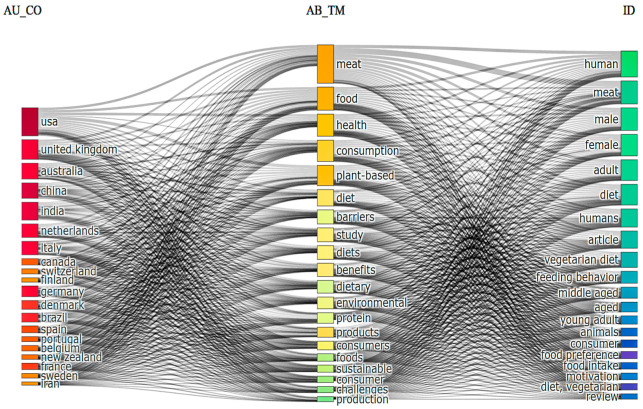
Three-Field Plot—Relationship between authors’ countries, abstract terms and Keywords Plus in the literature on plant-based diets. Source: Bibliometrix author data processing.

The Three-Field Plot presented in Figure 3 highlights the relationships among three key bibliographic components: automatically generated keywords (Keywords Plus-ID), together with frequent terms extracted from article abstracts (AB_TM), authors’ countries of affiliation (AU_CO). This ocular assessment permits an apprehension of how the texts on plant-based diets are configured and subjectively apportioned. It presents geographical disparities and preeminent conceptual orientations also.

Key terms such as “barriers”, “benefits”, “consumption”, “plant-based”, “health” and “diet” are fundamentally at the core of the figure because they accentuate the importance of these ideas in present-day scholarly discussion. The existence of the phrases “barriers” and “benefits” indicates a considered apprehension within the literature. This presence is simultaneous coupled with well-connected, indicating the barriers consumers face in adopting a plant-based diet and its perceived as well as documented benefits.

An analysis of interlinkages between the countries of affiliation of the authors, as well as between the terms extracted from the abstracts of the papers, depicts a strong contribution to research on plant-based diets by the United States, the United Kingdom, Australia, the Netherlands, China, and Italy. They are highly linked to basic notions such as “barriers”, “benefits”, “consumers”, “health” and “environment”, suggesting a keen interest in issues of public attitudes, cultural and economic barriers, and nutritional and environmental impacts of plant-based diet adoption.

For example, the regular pairing of the word “barriers” with countries such as the United States and the United Kingdom suggests that there is a dominant focus on behavioral and psychological factors, e.g., resistance to change, the influence of social norms, or unavailability of relevant information. In contrast, the frequent mention of the word “benefits” would appear to take place primarily in issues related to human health, food sustainability, and environmental footprint minimization. This difference is also supported by the analysis of additional keywords (column “Keywords Plus”), which habitually relates these terms to concepts of “consumer”, “motivation”, “eating behaviour”, “vegetarian diet”, “food preferences” and “sustainable”. As such, expert studies go further than the analysis of the characteristics of the food itself, tackling the perceived barriers and benefits of taking up such diets. This holistic approach confirms that the uptake of a plant-based diet is tackled in scientific literature through a multidimensional approach, where consumer behavior study and policy-making on sustainable food involve both barrier identification and benefit appreciation. The wide global distribution of this work reflects interest in sustainability worldwide and contributions are not only from countries with a long tradition of nutrition research, but also from emerging spaces in Asia and Eastern Europe, reflecting increased diversity and interdisciplinarity of this field of research.

**Figure 4:**
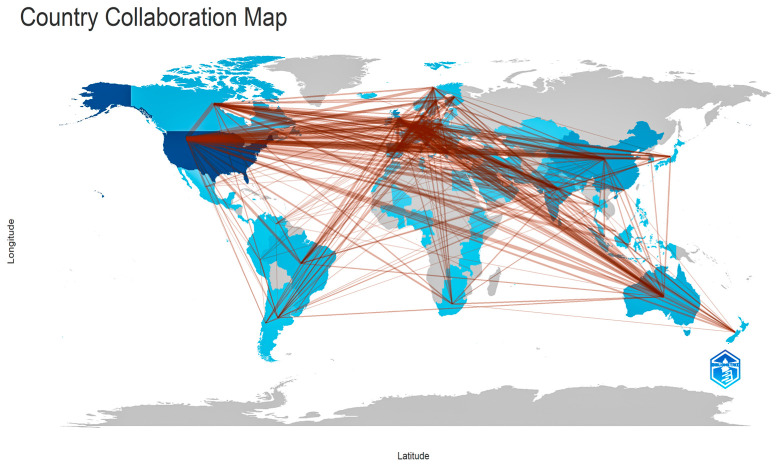
Map of international collaborations in plant-based diet research. Source: Bibliometrix author data processing. Shades of blue = number of publications (darker = higher output); grey = no publications; red lines = international collaborations.

The depiction of international collaborations between countries (Figure 4) furnishes an exhaustive depiction regarding scientific investigations into plant-based diets’ worldwide dynamics. This map highlights cooperative networks among countries, reflecting the academic linkage level and transnational alliances forged in this interdisciplinary domain.

Based on what the map indicates, the USA arises as being the central node in terms of scientific collaborations, creating relationships with a majority of countries that are involved within the research that was analyzed. This dominant position may be explained by a combination of factors, including a potentially higher institutional capacity, greater allocation of financial resources to research, and the country’s presumed role as a traditional scientific leader at the international level.

Concurrently, Europe presents itself as a thickly linked region since diverse countries, like Germany, the United Kingdom, France, Italy and the Netherlands, vigorously collaborate within a network. Academic institutions frequently research collectively, these regional as well as transatlantic links suggest. Furthermore, they are amenable toward knowledge interchange.

China, India, Japan, together with Australia, do actively participate in worldwide research networks representing the Asia-Pacific region substantially. People show increasing interest regarding sustainable nutrition and public health topics since those people are implicated. Environmental concerns along with accelerated urbanization are often linked in conjunction to them. Latin America (e.g., Brazil) and several portions of Africa (e.g., South Africa) are additionally present throughout the network, although with a reduced level of connectivity, which does indicate some large potential for broadened collaborations within regions located in the global south.

This map indicates that plant-based diet research constitutes a topic of universal interest from a cooperative standpoint. The presence of wide-ranging scientific networks favors the exchange of good practices, the development of common methodologies, and the comparability of results across regions. Concurrently, international organizations do collaborate as well as tackle global predicaments in an integrative fashion, such as climate change, food security, also sustainable food systems’ transition.

**Figure 5:**
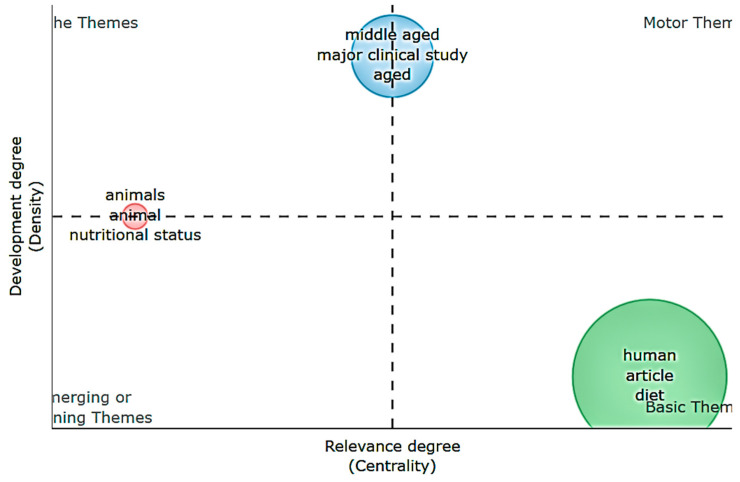
Thematic map of the main topics in the scientific literature on the transition to a plant-based diet. Source: Bibliometrix author data processing.

The thematic map presented in Figure 5 gives an overview of the main research themes identified in the literature on the transition to a plant-based diet. The chart is created based on the co-occurrence analysis of keywords and categorized according to the importance of the theme and the degree of development.

In the lower right quadrant, we find the basic themes, which include terms such as “human”, “article” and “diet”. These topics are widely covered in the literature and form the conceptual basis of the field. However, their relatively low density suggests that they are general, underdeveloped topics, and more in-depth research linking these concepts to public health, sustainability and consumer behavior is needed. In the top left quadrant are the specialty themes, with “middle aged”, “aged” and “major clinical” study themes. These reflect research directions that are well developed methodologically but of limited general relevance. This may suggest that there are very many clinical trials applied in the elderly population, suggesting that the benefits of plant-based diets are often analyzed in the context of aging and difficult-to-treat ailments of a certain age.

In the emerging and declining themes quadrant we find topics such as “animals”, “animal” and “nutritional status”. These themes are poorly developed, hence we can consider that research interest in these themes is low. Although “animals” might seem to be a secondary term, it is essential in analyzing the ethical and cultural barriers associated with the transition from an omnivorous to a plant-based diet. The ethical dimension of animal consumption is still poorly addressed in the literature. Also, the presence of the term “nutritional status” suggests that concerns about nutritional balance in plant-based diets are not yet systematically addressed or do not generate major research interest. Interestingly, none of the thematic clusters are in the top right quadrant, the quadrant corresponding to the driving themes. This absence suggests that the field of research on the transition to a plant-based diet is in the process of thematic consolidation, with no dominant direction representing the center of research. Given the increased interest in these studies through the number of articles, it is likely that research directions will shift towards more focused topics in the coming years.

**Figure 6:**
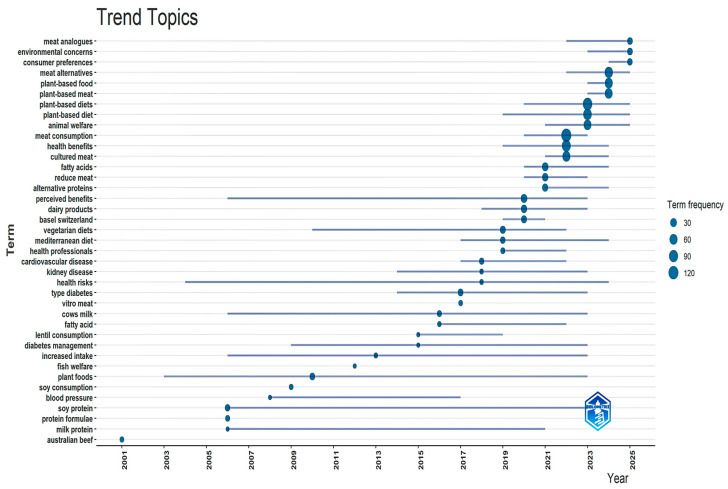
The evolution of topics of interest on plant-based food: benefits, barriers and emerging trends (2000–2024). Source: Bibliometrix author data processing.

A visual analysis concerning the frequency of relevant terms in the literature (2000–2024) (Figure 6) spotlights a thematic transition from discrete concerns respecting nutritional composition (e.g., ‘soy protein’, ‘protein formulae’) to a broader, integrative debate respecting perceived environmental benefits and public health.

Diminished likelihood of cardiopathy, type 2 diabetes, nephropathy and further persistent co-morbidities are among the most commonly referenced advantages, mirrored in the increased incidence of phrases such as “health benefits”, “cardiovascular disease”, “kidney disease” and “type diabetes”. Individuals utilize the expressions “vegetarian diets” as well as “Mediterranean diet” when they moderately or reductively ingest meat. Medical experts advocate these eating plans, thereby signaling factual confirmation of the dietary advantages.

On the other hand, perceived barriers impede individuals from extensively embracing plant-based comestibles. “Customer inclinations” and “comprehended advantages” constitute expressions depicting a disparity amid empirical substantiation and communal standpoints. This incongruity mirrors the definition of these expressions. Furthermore, apprehensions concerning health hazards coupled with the protein constitution of plant-derived selections (e.g., ‘soy protein’, ‘protein formulae’) imply a reticence to recognize nutritional sufficiency. The connection to animal derived commodities alongside apprehensions regarding the flavor or constitution of replacements (‘meat analogs’, ‘cultured meat’) emphasize these matters.

Across the span of 2020–2023, the investigation elucidates an increase in both the phrases “environmental concerns” and “animal welfare”. The increased appearance of terms such as “environmental concerns” and “animal welfare” in the 2020–2023 interval (see Figure 6) suggests that ethical and sustainability considerations are becoming more influential in shaping consumer behavior. Accordingly, value-driven and organized aspects, coupled with wholesome premises, propel the transition to plant-centric diets.

The bibliometric analysis carried out in the paper revealed that the studies present in the specialized literature do not sufficiently address the issue of benefits and barriers to adopting a plant-based diet. Also, another important point of our research was the identification of the benefits and barriers encountered by consumers in adopting a plant-based diet. An in-depth qualitative analysis of an extensive set of articles, selected not only from Scopus but also from other relevant academic sources (Google Scholar, PubMed), was conducted to include varied perspectives on the benefits and barriers associated with the transition to a plant-based diet. This stage aimed to critically interpret the scientific content, with a focus on nutritional, economic, ecological and cultural dimensions, thus providing a nuanced and integrative view of the investigated topic (Table 2 and Table 3).

**Table 2:** Overview of the benefits of plant-based product consumption as reported in the scientific literature.

Aspects	Benefits of Consuming Plant-Based Products	Citation
Nutritional	Plant-based diets offer significant nutritional benefits, contributing to metabolic health through high intakes of fiber, antioxidants, essential vitamins and minerals. They support weight management, reduce the risk of chronic diseases, and are aligned with food sustainability goals.	(Botnaru et al., 2025) [107], (Bunge et al., 2024) [58], (Craig and Mangels, 2009) [29], (Craig, 2010) [32], (Esquivel, 2022) [108], (Fehér et al., 2020) [2], (Hidalgo-Fuentes et al., 2024) [109], (Khalid et al., 2022) [19], (Marshall and Marinova, 2019) [31], (Patel et al., 2020) [110], (Pavlidou et al., 2023) [111], (Petrescu-Mag et al., 2025) [5], (Richter et al., 2015) [17], (Siegrist and Hartmann, 2019) [10], (Stavitz et al., 2025) [112], (Tso et al., 2021) [30], (Viroli et al., 2023) [56], (Yanni et al., 2023) [113], (Yu et al., 2023) [49], (Viere et al., 2021) [47]
Economic	Plant-based diets offer economic benefits at the individual level, by reducing the costs of tests, medical procedures or treatments for certain diseases. It also stimulates economic growth by developing plant-based food industries and promoting an inclusive and sustainable consumer environment. The development of the plant-based food industry is stimulating new opportunities in areas such as alternative protein production and food technology.	(Arpinon, 2024) [114], (Baliwati and Rusyda, 2024) [115], (Boukid, 2024) [116], (Fehér et al., 2020) [2], (Grabs, 2015) [54], (Lea et al., 2006) [117], (Masset et al., 2014) [53], (Pais et al., 2022) [55], (Singh et al., 2025) [20], (Viroli et al., 2023) [56]
Environmental	Switching to a plant-based diet has a number of environmental benefits, including significantly reduced greenhouse gas emissions and reduced water consumption compared to resource-intensive animal-based diets. In addition, adopting a plant-based diet can help conserve natural resources and protect biodiversity through sustainable agricultural practices. Animal agriculture is a major source of water contamination, and a plant-based diet reduces pressure on hydrological resources.	(Bobe et.al., 2025) [45], (Botnaru et al., 2025) [107], (Gibbs and Cappuccio, 2022) [15], (Ferrari et al., 2022) [11], (Kew et al., 2023) [6], (Kustar et al., 2021) [46], (Rodés-Bachs et al., 2024) [118], (Viere et al., 2021) [47], (Yu et al., 2023) [49]
Cultural	The adoption of this type of diet increasingly reflects a cultural shift, in which dietary diversity and ecological awareness are becoming central elements in modern eating practices.	(Arpinon, 2024) [114], (Bogueva and McClements, 2023) [119], (Botnaru et al., 2025) [107], (Shah and Thanki, 2024) [120], (Viroli et al., 2023) [56]

Plant-based diets offer nutritional, economic, environmental, and cultural benefits, increasingly justifying global adoption amid mounting social and ecological pressures.

### 4.1. Nutritional Benefits

Recent literature consistently highlights the nutritional strengths of well-planned plant-based diets. These diets emphasize the consumptions of fruits, vegetables, whole grains, legumes, nuts and seeds, which are rich in fiber, vitamins (A, C, E, folate), minerals (potassium, magnesium), antioxidants, phytonutrients, and unsaturated fats, while typically lower in saturated fat and cholesterol [58,107,108]. Such nutrient-dense profiles are associated with improved cardiovascular health, better lipid profiles, reduced inflammation, enhanced gastrointestinal function, and decreased risk of chronic diseases such as type 2 diabetes and certain cancers, such as colorectal, ovarian and breast [2,5,17,19,31,47].

A study conducted by Tso et al. [30] on the UK food market demonstrated that plant-based products contain greatly less saturated fat as well as energy furthermore provide a notably higher intake of dietary fiber, possibly leading to better diet quality in addition to the prevention of chronic diseases linked with unbalanced diets.

According to Craig and Mangels [29] and Craig [32], a properly designed plant-based diet can meet all macronutrient and micronutrient needs, including protein, iron, calcium, vitamin D, and vitamin B12 (the latter requiring supplementation or fortified sources). Furthermore, newer plant-based products, such as fortified milks and legume-based meats, often provide nutrient levels (e.g., protein, calcium, vitamins) comparable to those found in animal-based products, with the added benefit of lower saturated fat and higher fiber content [49,113].

Preparation techniques such as soaking, sprouting, fermenting, plus mild cooking (e.g., steaming) can increase nutrient bioavailability, conserve vitamins including phytonutrients, and improve nutritional results, notably when they are tailored toward varied cultural dietary patterns [56,109]. Fermented plant-based beverages contribute bioactive antioxidant as well as anti-inflammatory compounds, plus these do support metabolic together with immune health [109,111].

Several studies support the positive role of plant-based diets in promoting a healthy gut microbiota, enhancing microbial diversity and the production of short-chain fatty acids, which further contribute to immune and metabolic function [111]. Additionally, the intake of polyphenols and carotenoids has been linked to antioxidant, anti-inflammatory, and cognitive benefits [5].

From a behavioral perspective, plant-rich diets are associated with healthier eating habits and lower consumption of ultra-processed foods, while recent research shows that a plant-based diet contributes to better absorption of micronutrients such as iron, folic acid and magnesium, and improvements in long-term health indicators such as lower rates of hyperlipidemia and better antioxidant and vitamin K status [112,121].

### 4.2. Economic Benefits

The reviewed literature consistently highlights the economic benefits of adopting plant-based diets at both individual and societal levels. Singh et al. [20] pointed out in research that the plant-based food sector is booming and projections show that it could exceed $27 billion by 2030, largely due to the increasing consumer shift towards sustainable and health-oriented diets.

Widespread adoption of a plant-based diet could significantly reduce healthcare costs by lowering the prevalence of chronic diseases such as cardiovascular disease and type 2 diabetes, leading to significant savings in treatments, medicines and hospitalizations, while locally sourced plant-based foods such as legumes and cereals are more affordable than imported animal products, particularly benefiting low- and middle-income households; at the same time, although many consumers consider these foods to be economical and affordable, there are still misperceptions about the price of plant-based alternatives [114,115,117].

Studies show that plant-based diets can meet nutritional needs at the same or lower cost than omnivorous diets, without affecting diet quality Masset et al., [53], and the preparation of whole and minimally processed plant-based foods, especially when local and seasonal, proves to be an economically efficient choice [56]. In addition, the consumption of plant-based food also has economic value due to reduced environmental externalities, as it requires fewer natural resources, which generates long-term societal savings; these benefits can be included in sustainability and food systems accounting models, and businesses are encouraged to invest in plant-based supply chains, which can yield financial returns [2,54]. As this industry grows, it is expected to create new jobs and promote a fairer distribution of resources [116].

Pais et al. [55] illustrate how plant-based initiatives in public institutions, like community gardens and school menus, can reduce food procurement costs, enhance food security, and stimulate local economies.

### 4.3. Environmental Benefits

Plant-based diets are an effective solution for achieving environmental goals and protecting global ecosystems and have a considerable impact on environmental sustainability. They help reduce greenhouse gas emissions, save natural resources and preserve biodiversity. Regarding the Greenhouse Gas Emissions and Climate Impact, the research by Bobe et al. [45], Kew et al. [6] and Yu et al. [49] demonstrated that shifting from animal-based to plant-based foods can lower dietary greenhouse gas emissions by up to 50%. A study conducted by Kustar and Patiño-Echeverri [46] highlights that vegan diets can reduce land use by 50–86%, water consumption by 22–70% and greenhouse gas emissions by 21–70% compared to omnivorous diets.

Plant-based foods, such as legumes, cereals, fruits and vegetables, generate far fewer greenhouse gas emissions per calorie or gram of protein compared to meat or dairy, making the dietary transition to a plant-based diet a key strategy for climate change mitigation. In terms of land- and water-use efficiency, plant-based diets require much less arable land, thus offering the potential to free up space for reforestation and conservation [11]. These diets also have a much lower impact on water resources, which is particularly important for regions facing water scarcity [107]. Reducing livestock contributes significantly to preventing habitat destruction and species loss, and plant-based diets are essential for maintaining ecological balance and respecting planetary boundaries [15,118]. Viere et al. [47] emphasize the need to include environmental criteria in official protein assessment standards, suggesting that food choices should also be guided by environmental implications, not only nutritional parameters.

### 4.4. Cultural Benefits

The cultural benefits of adopting plant-based diets are complex and include both the preservation of culinary traditions and the emergence of new cultural values. These diets contribute to culinary diversification, foster ethical awareness and support cultural resilience through their ability to strengthen local communities and promote sustainable practices.

Plant-based diets reinforce Mediterranean, Asian and African culinary traditions, which have always emphasized legumes, grains and vegetables [56]. By promoting the use of seasonal and local ingredients, these diets help reconnect people with regional food identities and reduce dependence on industrialized and globalized food systems. They also increasingly reflect food choices based on ethical and identity-based values, especially among younger generations, which embrace principles such as care for both their own health and the environment, animal welfare and global justice, fostering cultural change, intergenerational dialog and global solidarity [119,122].

Another important aspect is stimulating culinary creativity, emphasized by Botnaru et al. [107], who show how mixing ingredients and cooking methods from diverse cultures, such as Indian, Middle Eastern and Latin American, has enriched global food traditions and supported cultural exchange. In addition, Shah and Thanki [120] explore how plant-based food practices can empower marginalized communities by restoring autonomy over food choices. Initiatives such as community gardens and local food systems help regain cultural agency and resist the influence of globalized food chains, especially in post-colonial and economically vulnerable areas.

At the same time, plant-based diets align with broader lifestyle trends such as minimalism, wellness, and slow living, which emphasize purposeful consumption and collective good, as Arpinon [114] has emphasized. As these diets gain visibility in the media and in policy, they contribute to evolving cultural norms around sustainability, health and ethical living.

**Table 3:** Overview of barriers to the consumption of plant-based products, as reported in the scientific literature.

Aspects	Barriers of Consuming Plant-Based Products	Citation
Nutritional	Adapting a regular diet to one based exclusively on plant-based products is often hindered by fear of nutritional deficiencies and lack of knowledge about properly planning a plant-based diet.	(Abe-Inge et al., 2024) [123], (Bunge et al., 2024) [58], (Craig et al., 2021) [124], (Gibbs and Cappuccio, 2022) [15], (Melina et al., 2016) [39], (Neufingerl and Eilander, 2021) [40], (Pavlidou et al., 2023) [111], (Rickerby and Green, 2024) [59], (Soh et al., 2025) [125], (Van Vliet et al., 2020) [79], (Viroli et al., 2023) [56]
Economic	Economic barriers to adopting a plant-based diet include the perception of high shelf costs for products. The accessibility and availability of plant-based products may be reduced in regions that do not have agri-food systems adapted to their production and distribution.	(Arpinon, 2024) [114], (Baliwati and Rusyda, 2024) [115], (Bunge et al., 2024) [58], (Chungchunlam et al., 2020) [126], (Gibbs and Cappuccio, 2022) [15], (Lea et al., 2006) [117], (Lusk and Norwood, 2016) [57], (Okop et al., 2019) [127], (Rickerby and Green, 2024) [59], (Viroli, Kalmpourtzidou and Cena, 2023) [56]
Environmental	Environmental barriers to adopting a plant-based diet include skepticism about the actual impact of diet on the environment. In some regions, plant-based foods may be difficult to obtain or expensive, affecting the accessibility and feasibility of the diet.	(Aimutis and Shirwaiker, 2024) [48], (Duque-Estrada and Petersen, 2023) [52], (Fehér et al., 2020) [2], (Gibbs and Cappuccio, 2022) [15], (Markowski and Roxburgh, 2019) [128], (Peydayesh et al., 2023) [50], (Rodés-Bachs et al., 2024) [118], (Rickerby and Green, 2024) [59], (Sproul et al., 2019) [51], (Viroli et al., 2023) [56], (Yu et al., 2023) [49]
Cultural	Cultural barriers to adopting a plant-based diet are deeply tied to social norms, food traditions, and collective perceptions that favor meat consumption, especially in Western societies.	(Gibbs and Cappuccio, 2022) [15], (Grasso and Jaworska, 2020) [22], (Purcărea et al., 2013) [81], (Petrescu et al., 2017) [82], (Rickerby and Green, 2024) [59], (Shah and Thanki, 2024) [120], (Viroli, Kalmpourtzidou and Cena, 2023) [56]

Although in scientific literature such consumption of plant food products is always emphasized with many positive points, transition of consumers to such diets is seldom accomplished without intense struggle, as it is driven by a range of complex motives, personal fears, prior experience, and regional socio-economic peculiarities.

### 4.5. Nutritional Barriers

The literature highlights a number of nutritional challenges associated with plant-based diets, which can be major barriers to their widespread adoption in the absence of adequate planning, nutrition education and political support. One of the most common concerns relates to inadequate intakes of essential nutrients such as vitamin B12, iron, zinc, calcium, iodine and omega-3 fatty acids. These substances are either less present in plant-based foods or are found in forms that are more difficult for the body to absorb [39,40]. Although some adaptations of the gut microbiota may support absorption, Pavlidou et al. [111] emphasize that these are not sufficient to fully compensate for deficiencies without a strategic nutritional approach or dietary fortification.

As for proteins, although plant proteins can meet the overall needs of the body, their amino acid profile is often less complete compared to animal proteins. For certain population groups, such as athletes or the elderly, either a higher intake or careful diversification of protein sources is needed [79,125]. In this regard, Bunge et al. [58] highlight the lack of knowledge among consumers on the correct combination of complementary plant proteins (e.g., legumes and cereals), especially in communities with low levels of nutritional literacy. In addition to these potential shortcomings, the circulation of misinformation and low levels of nutrition literacy contribute to fears of deficiency and distrust of fortified foods or supplements [59]. The cultural association between the idea of a “balanced diet” and the presence of animal protein discourages the transition to a predominantly plant-based eating pattern, even in the face of demonstrated health benefits [15]. Another important thing to note about the fact that many plant foods may contain major food allergens, which can make diet planning even more difficult [123].

Even though staple plant foods are often economically accessible, Viroli et al. [56] caution that nutrient-dense plant products-such as fortified plant drinks or meat substitutes-can be expensive or difficult to find, especially in disadvantaged regions. This lack of access limits the practical application of a nutritionally balanced plant-based diet. In addition, Pavlidou et al. [111] emphasize that individual variations in gut microbiota influence nutrient absorption capacity. In some cases, increased consumption of fiber or legumes may cause digestive discomfort, which may affect motivation and long-term adherence to a plant-based diet.

In order to overcome these barriers, educational interventions and communication strategies aimed at informing the population about the necessary dietary adjustments and supplements essential for maintaining a healthy plant-based diet should be implemented [59,124]. In addition, it is important to revise national dietary guidelines to support the transition to a plant-based diet, thereby ensuring the nutritional adequacy and sustainability of these diets [56].

### 4.6. Economic Barriers

Economic barriers to the adoption of plant-based diets are complex and vary considerably depending on the socio-geographical context. While there are many accessible plant-based food options, such as legumes, whole grains or local vegetables, perceptions of high costs, lack of access to fortified or convenient products, structural market limitations and lack of nutrition education contribute to limited and unequal uptake of these diets. Many consumers perceive plant-based diets-especially when they include organic or meat alternatives-as expensive. Even if staple foods are generally affordable, the association with premium products, such as vegan cheeses or fortified beverages, discourages people on a limited budget [57,117,126,127]. Low awareness of simple and affordable plant-based options reinforces the misperception that such a diet is financially inaccessible [58]. In economically deprived areas, even when local produce is available, fortified or processed options needed for a nutritionally complete diet are often hard to find or too expensive [115]. In addition, as Viroli et al. [56] point out, seasonal and geographical variations in access to fresh produce make it difficult for low-income households to maintain a consistent plant-based diet. In addition to these issues, structural barriers contribute to high prices for plant products. The lack of subsidies for plant-based agriculture, as well as little support for integrating these options into public institutions (such as school canteens or hospitals), limits their accessibility [114]. In the absence of public investment in production infrastructure, the plant-based sector fails to achieve the economies of scale enjoyed by the animal-based industry, which affects its competitiveness [15]. Another major obstacle is the lack of practical skills needed to adopt an economical plant-based diet. Rickerby and Green [59] point out that many consumers lack the basic knowledge of food budget planning or preparing plant-based meals, which often leads to over-consumption of ultra-processed, more expensive products and reinforces the idea that plant-based diets are elitist or impractical. In many societies, meat continues to be perceived as a symbol of prosperity and tradition, making it appear more “valuable” than plant-based alternatives, even when these are more affordable or healthier. Thus, cultural logic may override economic or nutritional logic [57].

### 4.7. Environmental Barriers

Although plant-based eating is recognized for its environmental benefits, its widespread adoption is hampered by a number of systemic and psychosocial barriers. One of the main difficulties is a lack of awareness of the environmental impact of food choices. Even among people concerned about sustainability, plant-based diets are often overlooked in favor of more visible issues such as transportation or energy consumption [15,59].

In addition to information deficits, cultural barriers play an important role. Deep-rooted perceptions that associate meat consumption with tradition and biological necessity reduce individuals’ willingness to change their diet [128]. These beliefs are often reinforced by a sense of ecological fatalism—the belief that individual choices have a negligible impact on global environmental problems—which discourages personal change initiatives. Furthermore, the current structure of food systems favors animal-based production through subsidies, infrastructure and well-developed supply chains, which limits access and scalability of plant-based options [118]. The lack of infrastructure for local and seasonal production of plant food leads to a dependence on imported or resource-intensive products, thus reducing the potential for sustainability. Geographical and seasonal access to fresh plant food is an additional barrier, especially in regions where agricultural diversity is low. In addition, increased demand for “superfoods” from vulnerable areas may lead to unsustainable agricultural practices and economic imbalance [56]. Consumer perceptions are affected by misleading marketing of ultra-processed plant-based products, promoted as organic despite the high environmental costs associated with their production. Such greenwashing practices erode consumer confidence and make it difficult to identify truly sustainable food options [2,59].

Although plant-derived products are promoted as an environmentally friendly alternative to animal products, the literature draws attention to the hidden impacts of industrial processes. The isolation of proteins from the plant matrix involves a succession of technical operations, such as mechanical milling, extraction and precipitation, which generate a considerable amount of waste [49]. The impact of processing is not limited to solid or liquid waste, but also extends to atmospheric emissions. Studies by Peydayesh et al. [50] and Sproul et al. [51] emphasize the significant contribution of this production phase to greenhouse gas emissions, particularly through the correlation between the amount of protein waste generated and carbon dioxide emissions. Thus, the perceived sustainability benefits of plant proteins are, in some cases, undermined by the energy and chemical intensity of refining processes. In the face of these challenges, the literature suggests a reorientation of industrial practices towards more resource-efficient extraction methods and, at the same time, the adoption of circular economy principles. This involves recovering the waste generated and reintegrating it into alternative value chains. This approach could not only reduce the environmental burden associated with the production of plant proteins, but also help to strengthen their legitimacy as a sustainable solution in the global food transition [48,52].

### 4.8. Cultural Barriers

Changing deep-rooted eating habits is often met with resistance, as food is a fundamental expression of cultural identity. For many people, changing their diet is not just an individual choice, but can be perceived as a break in social cohesion and continuity of traditions. Meat consumption has a strong cultural significance in many societies, symbolizing identity, social status and belonging, and is often an integral part of family rituals, celebrations or traditions. In this context, adopting a plant-based diet may be perceived as a threat to cultural heritage or personal identity [15,22]. Eating habits are also shaped by social norms and expectations, making plant-based diets sometimes seen as atypical or even deviant. Petrescu et al. [129] highlight that the pressure to conform to dominant, often meat-centered, norms can discourage the choice of alternative food options, especially in collective contexts such as family meals or social gatherings. In addition to social and cultural dimensions, perceptions of food satisfaction significantly influence acceptance of plant-based diets. Many consumers fear that meat-free meals would not be sufficiently nutritious or satisfying, both taste-wise and symbolically [59]. These concerns are often accompanied by a certain amount of skepticism or distrust, particularly in communities where meat diets are a source of collective pride and identity. In some cases, plant-based diets are perceived as foreign, elitist or externally imposed, which contributes to their rejection [81]. The public representation of these food choices also plays an important role. Plant foods are often underrepresented in the media and in mainstream culinary culture, and are sometimes associated with negative stereotypes, which reduces their attractiveness and cultural relevance [120]. Finally, cultural barriers frequently intersect with economic barriers. Viroli et al. [56] show that, depending on the context, plant-based diets can be perceived either as a necessity option and associated with poverty or as an unaffordable luxury, which sends contradictory and sometimes demobilizing messages to consumers.

## 5. Conclusions

The bibliometric analysis and thematic synthesis demonstrate a significant increase in scholarly engagement with plant-based dietary patterns over the past two decades, revealing the multifaceted nature of this expanding research field. The investigation illustrates the evolving academic community’s recognition of plant-based food as a complex phenomenon that intersects nutritional science, economic theory, environmental sustainability, and cultural studies. The analysis reveals a pronounced geographical concentration of research on plant-based diets in North America and Europe, with the United States maintaining its academic leadership in terms of both publication volume and scientific influence. The temporal evolution of research themes demonstrates a paradigm shift from initial concerns about the nutritional adequacy of plant-based foods to comprehensive examinations of public health outcomes, ecological sustainability, and ethical consumption patterns. This thematic progression reflects the maturation of the field from narrow nutritional surveys to holistic assessments of food systems.

The synthesized evidence confirmed the substantial multisectoral benefits of adopting plant-based diets. From a nutritional perspective, these dietary patterns demonstrate increased intake of dietary fiber, antioxidants, and essential micronutrients, correlating with a reduced risk of chronic disease and improved metabolic markers of health. The economic implications are manifested at both the individual and macroeconomic levels through reduced healthcare expenditures and the emergence of innovative food technology sectors, particularly alternative protein production systems. Environmental benefits include significant reductions in greenhouse gas emissions, water consumption, and agricultural land use, contributing significantly to climate change mitigation and biodiversity conservation strategies. Culturally, the adoption of plant-based diets promotes ethical consumption and environmentally responsible lifestyle choices. However, the analysis also identified substantial barriers to adoption, including limited product accessibility in certain regions, deeply rooted cultural preferences for animal protein consumption, particularly in Western societies, and persistent concerns about the nutritional adequacy of plant-based diets. Additional impediments include inadequate infrastructure for nutrition education and the widespread perception that individual dietary choices do not have a significant impact on the global environment.

The authors advocate for an integrated, interdisciplinary approach to address these multidimensional challenges. Policy recommendations include incorporating sustainability criteria into national dietary guidelines and implementing comprehensive nutrition education programs starting in early childhood. Industry stakeholders need to prioritize technological innovation to optimize the convergence of nutritional value, sensory quality, and economic affordability. The research community needs more empirical, culturally sensitive investigations that examine consumer behavior patterns and the effectiveness of policy interventions in diverse demographic contexts. The authors emphasize that effective transition strategies must include inclusive food policies, investments in sustainable agri-food infrastructure, support for plant-based innovation, and integration of nutrition education into formal school curricula.

This analysis also has some limitations, as the exhaustive coverage of the global literature may be limited due to the fact that the bibliometric analysis is performed exclusively based on data obtained from the Scopus platform. In addition, the search terminology designation may have omitted some relevant studies that address the topic in indirect ways.

Further research should focus on analyzing real-world consumer perceptions in different cultural contexts and systematically assessing the impact of regulatory initiatives on the acceptance of plant-based diets.

## Figures and Tables

**Table 1:** Number of articles published between 1957 and 2025.

Publication Period of Scientific Articles	Number of Published Scientific Articles	Percentage of Total Articles Published
1957–1990	122	3.75%
1991–2005	320	9.86%
2006–2015	461	14.20%
2016–2020	657	20.24%
2021–2023	1039	32.01%
2024–2025	646	19.90%
Total	3245	-

## Data Availability

No new data were created or analyzed in this study. Data sharing is not applicable to this article.
